# Multifocal and extrafacial chronic violaceous plaques

**DOI:** 10.1016/j.jdcr.2025.10.023

**Published:** 2025-10-24

**Authors:** Maria M. Hornberger, William C. Schaffenburg, Paul S. Hahn

**Affiliations:** a Department of Dermatology, San Antonio Uniformed Services Health Education Consortium, JBSA-Lackland, Texas; b Departments of Dermatology & Pathology, San Antonio Uniformed Services Health Education Consortium, JBSA-Lackland, Texas

**Keywords:** benign, cryotherapy, dapsone, extrafacial, granuloma faciale, grenz zone, head and neck, multifocal, pulsed dye laser, tacrolimus

## Case description

**Fig 1:**
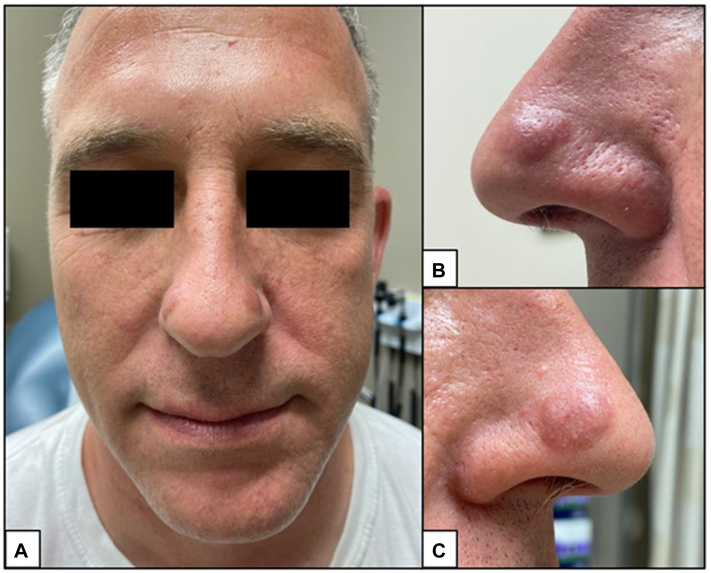
Clinical examination of well-demarcated, red-brown to violaceous blanchable plaques located symmetrically on the bilateral nasal alae **(A)**, with a closer view of the left **(B)** and right **(C)** side demonstrating dilated follicular ostia and follicular plugging.

**Fig 2:**
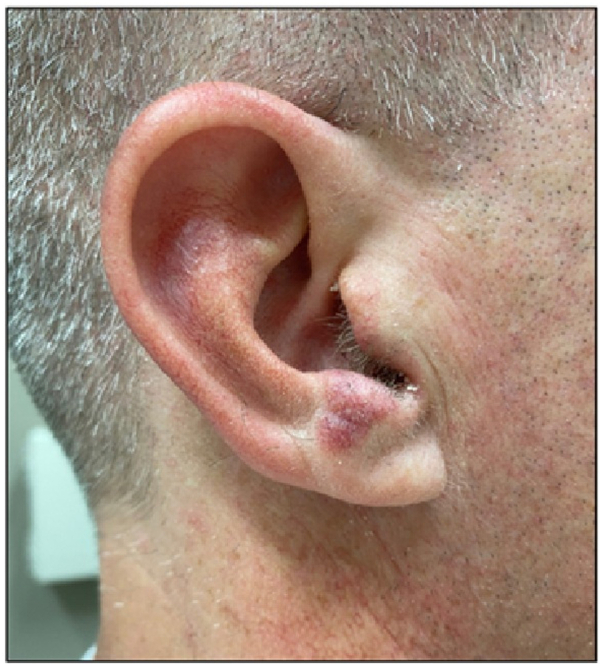
Clinical examination of well-demarcated, red-brown to violaceous blanchable plaques with overlying telangiectasias located on the right antitragus, scapha, and helix.

**Fig 3:**
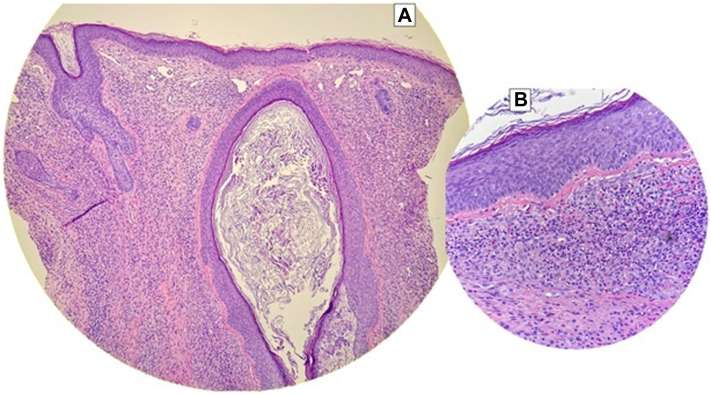
A punch biopsy from the left nasal ala showing a diffuse inflammatory dermal infiltrate, prominent sparing of the upper papillary dermis, and a dilated follicle **(A)**. A higher power view of the dermal infiltrate showing numerous lymphocytes, neutrophils, eosinophils, and plasma cells **(B)**. (H&E, original magnifications ×100 and ×400).

A 43-year-old healthy male presented for evaluation of asymptomatic “lumps” on the nose present for at least 1 year. Physical examination revealed multiple well-demarcated, red-brown to violaceous blanchable plaques with prominent follicular openings located symmetrically on the bilateral nasal alae (Fig 1). No overlying secondary skin changes such as scale were seen. On further examination, smaller but similar appearing lesions were identified on the bilateral antitragi, right scapha, and right helix, ranging in size from 6 × 5 mm to 15 × 11 mm (Fig 2). The remainder of the physical exam and review of systems was unremarkable. The differential diagnosis included lupus pernio, sarcoidosis, cutaneous lymphoma, tumid lupus erythematosus, or another granulomatous disorder. A punch biopsy was performed. Histological examination demonstrated a diffuse mixed inflammatory dermal infiltrate, composed of numerous lymphocytes, neutrophils, eosinophils, and plasma cells, as well as a prominent sparing of the upper papillary dermis (Fig 3).

**Question: What is the best diagnosis?**

A. Cutaneous lymphoma
B. Granuloma faciale (GF)
C. Lupus pernio
D. Sarcoidosis
E. Tumid lupus erythematosus

## Discussion

Answer: **B**. GF

GF is an uncommon, benign, chronic inflammatory dermatosis of uncertain pathogenesis, thought to be a localized form of skin-limited small vessel vasculitis.^1^, ^2^, ^3^ It classically presents as a solitary, asymptomatic, red-brown or violaceous plaque on the face, often with follicular accentuation and superficial telangiectasias, and without associated systemic disease.^1^^,^^2^ GF has a slight predilection for middle-aged Caucasian males and is usually asymptomatic, although can be mildly pruritic.^2^ Characteristic histologic features of GF include a dense mixed inflammatory infiltrate separated from the overlying epidermis by a narrow grenz zone of uninvolved dermis, in addition to telangiectasia.^4^

GF is often clinically misdiagnosed, especially if a classic lesion presents in an atypical way, as in our patient.^4^ Developing multiple lesions in GF is uncommon (38% of cases) and extrafacial involvement, such as the ears, is rare (1% to 6% of cases).^4^ Ultimately, biopsy is recommended to rule out other skin diseases with a similar appearance and confirm the diagnosis.^1^ The chronic nature of GF and its associated cosmetic disfigurement can be distressing to patients, which typically leads to seeking care.^2^ Unfortunately, due to the incomplete understanding of underlying pathological mechanisms, developing effective therapeutic interventions for GF has been challenging.^3^

Management of GF can be difficult with variable clinical responses, to include risk of scarring and recurrence.^1^ It is important for dermatologists to be familiar with the multiple topical, systemic, and mechanical treatment regimens available for GF. Based on published treatment successes, a 2018 systematic review recommended starting treatment with topical tacrolimus 0.1% ointment twice daily, with consideration of topical dapsone gel.^1^ Cryotherapy is widely accessible and inexpensive, though with mixed reports on effectiveness.^1^^,^^5^ Systemic dapsone is normally well-tolerated at 100 mg daily, however, thorough counseling is required regarding its potential severe side effects and related lab monitoring.^1^ For drug-resistant GF, pulsed dye laser is preferred if available.^1^ Our patient noted initial then diminished improvement with tacrolimus ointment over the course of 1 year. During this time, intralesional triamcinolone was attempted for the thick nasal lesions with limited benefit. Our patient later reported significant improvement after transitioning to topical dapsone and pulsed dye laser.

## Conflicts of interest

None disclosed. The views expressed are those of the author(s) and do not reflect the official views or policy of the Department of War or its components.
